# High-Throughput Screening and Characterization of Phenolic Compounds in Stone Fruits Waste by LC-ESI-QTOF-MS/MS and Their Potential Antioxidant Activities

**DOI:** 10.3390/antiox10020234

**Published:** 2021-02-04

**Authors:** Yili Hong, Zening Wang, Colin J. Barrow, Frank R. Dunshea, Hafiz A. R. Suleria

**Affiliations:** 1 School of Agriculture and Food, Faculty of Veterinary and Agricultural Sciences, The University of Melbourne, Parkville, VIC 3010, Australia; yilhong@student.unimelb.edu.au (Y.H.); zeningw1@student.unimelb.edu.au (Z.W.); fdunshea@unimelb.edu.au (F.R.D.); 2 Centre for Chemistry and Biotechnology, School of Life and Environmental Sciences, Deakin University, Waurn Ponds, VIC 3217, Australia; colin.barrow@deakin.edu.au; 3 Faculty of Biological Sciences, The University of Leeds, Leeds LS2 9JT, UK

**Keywords:** fruit waste, stone fruits, phenolic compounds, LC-ESI-QTOF-MS/MS, HPLC-PDA

## Abstract

Stone fruits, including peach (*Prunus persica* L.), nectarine (*Prunus nucipersica* L.), plum (*Prunus domestica* L.) and apricot (*Prunus armeniaca* L.) are common commercial fruits in the market. However, a huge amount of stone fruits waste is produced throughout the food supply chain during picking, handling, processing, packaging, storage, transportation, retailing and final consumption. These stone fruits waste contain high phenolic content which are the main contributors to the antioxidant potential and associated health benefits. The antioxidant results showed that plum waste contained higher concentrations of total phenolic content (TPC) (0.94 ± 0.07 mg gallic acid equivalents (GAE)/g) and total flavonoid content (TFC) (0.34 ± 0.01 mg quercetin equivalents (QE)/g), while apricot waste contained a higher concentration of total tannin content (TTC) (0.19 ± 0.03 mg catechin equivalents (CE)/g) and DPPH activity (1.47 ± 0.12 mg ascorbic acid equivalents (AAE)/g). However, nectarine waste had higher antioxidant capacity in ferric reducing-antioxidant power (FRAP) (0.98 ± 0.02 mg AAE/g) and total antioxidant capacity (TAC) (0.91 ± 0.09 mg AAE/g) assays, while peach waste showed higher antioxidant capacity in 2,2′-azino-bis-(3-ethylbenzothiazoline-6-sulfonic acid (ABTS) assay (0.43 ± 0.09 mg AAE/g) as compared to other stone fruits waste. Qualitative and quantitative phenolic analysis of Australian grown stone fruits waste were conducted by liquid chromatography coupled with electrospray-ionization quadrupole time-of-flight mass spectrometry (LC-ESI-QTOF-MS/MS) and HPLC-photodiode array detection (PDA). The LC-ESI-QTOF-MS/MS result indicates that 59 phenolic compounds were tentatively characterized in peach (33 compounds), nectarine (28), plum (38) and apricot (23). The HPLC-PDA indicated that *p*-hydroxybenzoic acid (18.64 ± 1.30 mg/g) was detected to be the most dominant phenolic acid and quercetin (19.68 ± 1.38 mg/g) was the most significant flavonoid in stone fruits waste. Hence, it could be concluded that stone fruit waste contains various phenolic compounds and have antioxidant potential. The results could support the applications of these stone fruit wastes in other food, feed, nutraceutical and pharmaceutical industries.

## 1. Introduction

Food waste is one of the main challenges and a world-wide problem. It may occur during the whole food supply chain which is directly or indirectly related to producers, retailers and ultimate consumers [1]. According to the estimation of Edwards and Mercer [2], 44 million tons of food is wasted in Australia each year. It has also been reported that 25–40% of food is wasted throughout the food supply chain [3]. Fruit injuries, bruising, over-ripening during food transportation and storage are also some of main issues of food waste [1,4]. In addition, rejected foods are also one of the main types of food waste; most consumers are generally reluctant to choose imperfect foods in terms of shape, color, size, appearance and freshness [5]. This fruit waste is rich in moisture content and biodegradable ingredients; it can produce unbearable gas and bacteria, which leads to greenhouse effect and plague [6]. In order to reduce these impacts, extraction of phytochemicals from different fruit waste streams has been one of the recent focuses [7].

Stone fruits are rich in phytochemicals and are usually consumed directly or processed into food products including jam, juices and so on [8]. Stone fruits are the members of *Prunus*, which includes peach (*Prunus persica* L.), nectarine (*Prunus nucipersica* L.), plum (*Prunus domestica* L.) and apricot (*Prunus armeniaca* L.) [9]. They consist of a thin outer layer (epicarp), edible flesh (mesocarp) and a hard stone (endocarp) which encloses the seed in the center of the fruit [10]. Stone fruits contain various bioactive compounds, which can be classified as carotenoid, vitamin and phenolic and thiol compounds, and may have potential antioxidant and anticancer activities [11]. For example, previous studies have extracted and identified more than 30 phenolic compounds from peaches [12]. Presence of these diverse bioactives in stone fruits is attractive to consumers and stone fruits have a high sales volume in the market [8], which indicates that utilization and repurposing of their waste is one of the smartest ideas to improve the circular economy and food sustainability.

Phenolic compounds can be widely found in plants, and consist of an aromatic ring with one or more hydroxyl substituents [13,14]. So far, there are more than 8000 kinds of phenolic compounds which have been identified in plants, including flavonoids, phenolic acids, tannins etc. [15]. Although the specific metabolic mechanism of phenolic compounds is unclear, previous studies reported that phenolic compounds have antioxidant, antibacterial and anticancer properties [16,17,18]. Previously, Zerva, et al. [19] confirmed that peach waste is rich in carotenoids and phenolic compounds. Gil, et al. [20] argued that peach waste contains a high concentration of β-carotene, ascorbic acid and phenolic compounds; furthermore, the antioxidant activity is mainly attributed to phenolic compounds. Previously, Liu, et al. [8] demonstrated that peach peels have higher levels of antioxidant activities than peach flesh because of their higher phenolic content. Michalska, et al. [21] found that the phenolic compounds in the pomace of plum are mainly procyanidin and catechin. Furthermore, different stone fruits contain different bioactive compounds; for example, the phenolic profile in plums and peaches varies greatly [22]. In addition, the composition of phenolics also changes during the maturity of stone fruits [23]. Therefore, it is necessary to characterize and quantify the phenolic compounds in stone fruits.

The antioxidant activity of phenolic compounds can be measured by scavenging free radicals using in vitro assays which include the 2,2’-diphenyl-1-picrylhydrazyl (DPPH) assay, ferric reducing-antioxidant power (FRAP) assay and 2,2′-azino-bis-(3-ethylbenzothiazoline-6-sulfonic acid) (ABTS) radical cation decolorization assay [24,25]. The phenolic content may vary depending on the extraction method, the material, the solvent and the environment [26]. Methanol, ethanol, acetone, ethyl acetate and aqueous mixtures of these solvents are commonly used for extraction [27]. Liquid chromatography coupled with electrospray-ionization quadrupole time-of-flight mass spectrometry (LC-ESI-QTOF-MS/MS) can be applied to determine the phenolic profile in rejected stone fruits with high accuracy. High-pressure liquid chromatography (HPLC) in combination with a photodiode array detector (PDA) is used to quantify the particular phenolic compounds in fruits and vegetables [28]. These two techniques can be considered to qualify and quantify the phenolic compounds in stone fruits waste. Previously, Wu, et al. [29] characterized some major phenolic compounds including kaempferol, gallic acid and (+)-catechin in one of the peach cultivars (Xiahui-8) using LC-MS in China. Sójka, et al. [30] also identified some phenolic compounds in dark blue plum including anthocyanins, neochlorogenic acid and chlorogenic acid in Poland. However, there is limited research published on Australian grown stone fruits and especially rejected stone fruits waste.

In this research, the potential antioxidant activity of Australian grown stone fruits waste including peach, nectarine, plum and apricot was examined. The objectives of this study were to extract the phenolic compounds from stone fruits waste; measure the total phenolic content (TPC), total flavonoid content (TFC), and total tannin content (TTC); analyzes the antioxidant activity by using DPPH, FRAP, ABTS and total antioxidant capacity (TAC) assays; characterize the phenolic compounds by LC-ESI-QTOF-MS/MS analysis and quantify from stone fruits waste using HPLC-PDA analysis. The outcome of our study may provide possibilities for the utilization of stone fruits waste in other food, feed and pharmaceutical streams.

## 2. Materials and Methods

### 2.1. Chemical and Reagents

Most of the chemicals used for phenolics extraction and characterization in this research were analytical grade and purchased from Sigma-Aldrich (Castle Hill, NSW, Australia). Folin-Ciocalteu reagent, aluminum chloride hexahydrate, 2,2’-diphenyl-1-picrylhydrazyl (DPPH), 2,4,6-tripyridyl-s-triazine (TPTZ), 2,2′-azino-bis-(3-ethylbenzothiazoline-6-sulfonic acid), gallic acid, ascorbic acid, quercetin, vanillin, catechin, potassium persulfate and HCl were purchased from Sigma-Aldrich (St. Louis, MO, USA). Ethanol, sodium carbonate, sulfuric acid, sodium acetate, acetic acid and ferric chloride (Fe[III]Cl_3_·6H_2_O) were purchased from the Thermo Fisher (Scoresby, Melbourne, VIC, Australia). HPLC grade methanol, acetonitrile and acetic acid were purchased from Sigma-Aldrich (St. Louis, MO, USA). Phenolic acid and flavonoid standards, including caffeic acid, chlorogenic acid, gallic acid, *p*-hydroxybenzoic acid, protocatechuic acid, catechin, epicatechin, epicatechin gallate, kaempferol and quercetin were purchased from Sigma-Aldrich (St. Louis, MO, USA).

### 2.2. Sample Preparation

Stone fruit waste samples used in the proposed research project were mostly rejected by the customers due to their low-grade quality in terms of shape, color, size, appearance, freshness, injuries and over ripeness but were not rotten. Samples of 2–3 kg of each stone fruits waste including peach (*Prunus persica* L.), nectarine (*Prunus nucipersica* L.), plum (*Prunus domestica* L.) and apricot (*Prunus armeniaca* L.) were collected from a local retail market in Melbourne, Australia. After removing the seeds and peels, samples were cleaned and crushed into small pieces, and prepared for extraction within 1–2 h. Pulps were blended (1.5-L blender, Russell Hobbs Classic, model DZ-1613, Melbourne, VIC, Australia) into slurry, stored in silver flat Ziplock aluminum foil—vacuum sealing bags (Best supply, NSW, AU) and were kept at −20 °C for 2–3 weeks for further analysis.

### 2.3. Extraction of Phenolic Compounds

Extracts were prepared with ethanol (70%, 20mL) by modifying the protocol of Gu, et al. [31]. In short, extracts were shaken over 12 h in a shaking incubator (ZWYR-240, Labwit, Ashwood, Victoria, Australia) at 120 rpm, 4 °C and then centrifuged (ROTINA 380R centrifuge, Hettich, Victoria, Australia) at 5000 rpm for 15 min. The supernatant was collected and stored at −20 °C for further analysis.

### 2.4. Estimation of Phenolic Compounds and Antioxidant Assays

TPC, TFC and TTC were determined for phenolic compounds estimation, while DPPH, FRAP, and ABTS were measured for antioxidant capacity. All the assays were performed using our previously modified method of Tang, et al. [32] in triplicate. The data were obtained by the Multiskan^®^ Go microplate reader (Thermo Fisher Scientific, Waltham, MA, USA). 

#### 2.4.1. Determination of Total Phenolic Content (TPC)

The TPC of stone fruits was determined by using the method of Severo, et al. [33] with modification. An amount of 25 µL Folin-Ciocalteu reagent (1:3 diluted with water) and 200 µL water was added to 25-µL extracts in triplicate in 96-well plates (Corning Inc., Midland, NC, USA) and incubated for 5 min at room temperature. Then, 25 µL of 10% (w/w) sodium carbonate was supplied to basify the mixture. After incubating at 25 °C for 60 min, the absorbance was determined at 765 nm with a spectrophotometer plate reader (Thermo Fisher Scientific, Waltham, MA, USA). The TPC was expressed as mg of gallic acid equivalent (GAE) per gram fresh weight (mg GAE/g fw) of the sample using the calibration curve of gallic acid standard (0–200 µg/mL).

#### 2.4.2. Determination of Total Flavonoids Content (TFC)

The TFC in stone fruits was determined by the modified aluminum chloride method of Gouveia and Castilho [34]. An amount of 80 µL of 2% (*w*/*v*) aluminum chloride ethanolic solution and 120 µL of 50 mg/mL sodium acetate solution were applied to 80 µL stone fruits extract in a 96-well plate, followed by incubation at room temperature in a dark room for 2.5 h. The absorbance was measured at 440 nm. The TFC was expressed as mg of quercetin equivalent (QE) per gram fresh weight (mg QE/g fw) of the sample using the calibration curve of prepared with quercetin with concentrations ranging from 0 to 50 µg/mL.

#### 2.4.3. Determination of Total Tannins Content (TTC)

Based on the method of vanillin and the *p*-dimethylaminocinnamaldehyde method of Stavrou, et al. [35], TTC was determined by mixing 150 µL of 4% (*w*/*v*) methanolic vanillin solution with a 25-µL diluted sample. Then, 25 µL of 32% (*v*/*v*) sulfuric acid in methanol was supplied to the mixture in a 96-well plate. The absorbance was measured at 500 nm after incubating at 25 °C for 15 min. The TTC was expressed as mg of catechin equivalent (CE) per gram fresh weight (mg CE/g fw) of the sample using the calibration curve of catechin (0–1000 µg/mL).

#### 2.4.4. 2,2′-Diphenyl-1-picrylhydrazyl (DPPH) Antioxidant Assay

The DPPH free radical scavenging capacity was determined by modifying the method of Sogi, et al. [36]. A 40-µL extract was added to 260 µL of 0.1-mM DPPH radical methanol solution in a 96-well plate, followed by shaking vigorously. Then, the mixture was incubated for 30 min at 25 °C. The absorbance was determined at 517 nm. The DPPH free radical scavenging capacity was expressed as mg of ascorbic acid equivalent (AAE) per gram fresh weight (mg AAE/g fw) of the sample using the calibration curve of ascorbic acid (0–50 µg/mL).

#### 2.4.5. Ferric Reducing-Antioxidant Power (FRAP) Assay

The FRAP was assayed with a modified method of Sogi, et al. [36]. The FRAP method evaluates the capacity of a material to reduce iron in the Fe^3+^–TPTZ complex (ferric-2,4,6-tripyridyl-s-Triazine) into Fe^2+^–TPTZ. The FRAP reagent was made from 20 mM FeCl_3_ solution, 10 mM TPTZ (2,4,6-tripyridyl-s-triazine) solution and 300 mM sodium acetate solution with a volume ratio of 1:1:10. An amount of 20 µL of sample was added to 280 µL FRAP reagent in a 96-well plate and incubated at 37 °C for 10 min. The absorbance was determined at 593 nm. The FRAP was expressed as mg of ascorbic acid equivalent (AAE) per gram fresh weight (mg AAE/g fw) of the sample using the calibration curve prepared from ascorbic acid with concentrations ranging from 0 to 50 µg/mL.

#### 2.4.6. 2,2′-Azino-bis-(3-ethylbenzothiazoline-6-sulfonic acid) (ABTS) Radical Scavenging Assay

The ABTS radical scavenging capacity is calculated by using ABTS radical cation decolorization assay using the protocol of Peng, et al. [37]. ABTS cations were generated by a mixture of 5 mL of 7 mmol/L of ABTS solution with 88 µL of a 140-mM potassium persulfate solution, which was incubated at 25 °C for 16 h in a dark area. The ABTS^+^ solution was then diluted with ethanol to obtain an initial absorbance of 0.70 at 734 nm. After that, 10 µL of the stone fruits sample was applied to 290-µL prepared ABTS^+^ solution in a 96-well plate and incubated at room temperature for 6 min in the dark. After the incubation, the absorbance was measured at 734 nm. The standard curve is also constructed by using ascorbic acid solution. The ABTS radical scavenging capacity was expressed as mg of ascorbic acid equivalent (AAE) per gram fresh weight (mg AAE/g fw) of the sample using the calibration curve prepared from ascorbic acid (0–150 µg/mL).

#### 2.4.7. Total Antioxidant Capacity (TAC)

The TAC was estimated by modifying the phosphomolybdate method of Prieto, et al. [38]. Phosphomolybdate reagent was prepared by mixing H_2_SO_4_ (0.6 M), sodium phosphate (0.028 M) and ammonium molybdate (0.004 M). A 40-µL extract was applied to 260 µL of phosphomolybdate reagent in the 96-well plate. The absorbance was measured at 695 nm after incubating at 95 °C for 10 min and cooling down to the room temperature. The TAC was expressed as mg of ascorbic acid equivalent (AAE) per gram fresh weight (mg AAE/g fw) of the sample using the calibration curve of ascorbic acid (0–200 µg/mL).

### 2.5. LC-ESI-QTOF-MS/MS Analysis

LC-ESI-QTOF-MS/MS characterization of the phenolic compounds in stone fruits waste was performed by Agilent 1200 series HPLC (Agilent Technologies, CA, USA) equipped with an Agilent 6520 Accurate-Mass Q-TOF LC/MS (Agilent Technologies, CA, USA) by following the protocol of Suleria, et al. [39]. The separation of compounds is achieved by using Synergi Hydro-RP 80A LC reverse phase column with an internal diameter of 250 mm × 4.6 mm and particle size of 4 µm (Phenomenex, Torrance, CA, USA). The Phenomenex C18 ODS guard column with an internal diameter of 4.0 × 2.0 mm is used to protect the column. The mobile phase A consisted of acetic acid/water solution (2:98, v/v), whereas mobile phase B was composed of acetonitrile/acetic acid/water (100:1:99, v/v/v). Mobile phases A and B were degassed at 25 °C for 15 min. The flow rate was set to be 0.8 mL/min and the injection volume of each sample was 6 µL. Gradient elution conditions were set by a mixture of mobile phase A and B as follows: 0–20 min, 10% B; 20–30 min, 25% B; 30–40 min, 35% B; 40–70 min, 40% B; 70–75 min, 55% B; 75–77 min, 80% B; 77–79 min, 100% B; 79–82 min, 100% B; 82–85 min, 10% B. The column was equilibrated for 3 min between each two injections.

For MS/MS, electrospray ionization (ESI) was utilized in operating both negative and positive ion modes. The mass spectrometry conditions were performed as follows: the nebulizer gas pressure was 45 psi, the nitrogen gas temperature was 300 °C with a 5 L/min flow rate, while the sheath gas temperature was 250 °C with an 11 L/min flow rate. The capillary and nozzle voltage were, respectively, set at 3.5 kV and 500 V. The mass spectra were obtained over the m/z range of 50–1300 amu with collision energy (10, 15 and 30 eV) for fragmentation. Data collection and analysis were performed using Agilent LC-MS-QTOF MassHunter data acquisition software version B.03.01.

### 2.6. HPLC-PDA Analysis

HPLC-PDA was carried out by using the method of Ma, et al. [40] to quantify the targeted phenolic compounds in stone fruits samples, which was performed with Agilent 1200 series HPLC (Agilent Technologies, CA, USA) equipped with a photodiode array (PDA) detector. Column and LC conditions were maintained as described above in LC-ESI-QTOF-MS/MS analysis except the sample injection volume is changed to 20 µL. The PDA detector is used to detect sample compositions under 280 nm, 320 nm, and 370 nm for the identification of hydroxybenzoic acids, hydroxycinnamic acids and flavonol groups, respectively. Individual phenolic compounds were quantified according to the calibration curves generated from standards. Results were expressed as µg/g of the sample. Data acquisition and analysis were performed using Agilent LC-ESI-QTOF-MS/MS MassHunter data acquisition software version B.03.01.

### 2.7. Statistical Analysis

Results were presented as mean ± standard deviation (SD) of triplicate experiments. One-way analysis of variance (ANOVA) was used to test whether there are significant differences between mean values of different samples, followed by Tukey’s honestly significant differences (HSD) multiple rank test at *p* < 0.05, which was carried out by using Minitab Statistical software for Windows Version 19.0 (Minitab, LLC, State College, PA, USA).

## 3. Results and Discussion

### 3.1. Phenolic Compounds Estimation (TPC, TFC and TTC)

Stone fruits have been reported to be rich in phenolic compounds [20,21]. In this research, the phenolic content in four Australian grown stone fruits’ waste including peach, nectarine, plum and apricot were determined by TPC, TFC and TTC (Table 1). Plum waste and apricot waste presented a higher phenolic content among all the samples, since plum waste showed a significant higher TPC and TFC, and apricot waste displayed a higher TTC than the others (*p* ≤ 0.05).

In terms of TPC, all the samples were significantly different from each other (*p* ≤ 0.05). Plum waste (0.94 ± 0.07 mg GAE/g) contained the highest concentration of phenolic compounds, followed by apricot, peach and nectarine. The previous results [41] also showed that the TPC of Serbian grown *“Cacanska secer”* plum is higher than *“J. H. Hale”* peach and *“Caldesi”* nectarine. This may be due to the difference in phenolic composition in different fruits. Since the skin of stone fruit is usually not eaten by humans. However, previous study has described that the peel of Chinese grown peach, which is *Hujingmilu* cultivar (79.14 ± 4.81 mg GAE/100g), contains more phenolic compounds than peach flesh (25.28 ± 0.96 mg GAE/100g) [8]. Furthermore, compared with the previous study with TPC of Serbian grown plum, our data were slightly lower [41]. It has been reported that total phenolic content varies within cultivars [8]; the lower phenolic concentration in our study may suggest that Australian grown stone fruits contain less phenolic compounds as compared to Serbian grown stone fruits. 

In TFC, plum waste (0.34 ± 0.01 mg QE/g) also showed significantly (*p* ≤ 0.05) higher concentration among others; however, there was no significant difference between peach and nectarine. Previously, it has been reported that TFC of north-western Iranian grown plums ranged from 16.06 to 35.81 mg QE/100 g, which was almost similar to our results [42]. However, compared with fresh Iranian peach, including *“Zoodras”*, *“Kosari”*, *“Haj-kazemi”*, *“Tak-daneh”*, *“Anjiri-ye-khouni*” and *“Zaferani”*, our TFC results were slightly lower [43]. This may suggest that stone fruits waste contain less flavonoids as compared to fresh fruits. It might be due to conversion of parent flavonoids into other metabolites. Fruit maturity is also one of the important factors, flavonoid content decrease significantly (around 40%) during ripening [44]. Rejected fruits are mostly over-ripen; therefore, they may have less flavonoids as compared to fresh stone fruits.

As for TTC, apricot waste contained the highest tannins (0.19 ± 0.03 mg CE/g). There was non-significant difference between the tannin content of peach and plum (*p* ≤ 0.05). Compared with the previous research on apricot and *“Papaz”* plum growing in Turkey, our samples showed higher tannin content. It has been revealed that tannin concentration varies from varieties, geographical origin and environmental conditions [45]. In addition, the fruit storage conditions have a significant impact on tannin content. Peaches stored at lower temperatures (0–2 °C) retained more phenolic compounds [46], long storage duration can also reduce phenolic compounds [46]. Moreover, tannin concentration may also be affected by growing conditions, agronomical practices and water availability. Under water scarcity and stress condition, the concentration of phenolic compounds normally increased because of the plant defense mechanism [47].

### 3.2. Antioxidant Activities Estimation (DPPH, FRAP, ABTS and TAC)

The antioxidant activities were determined by DPPH, FRAP, ABTS and TAC, which are the most preferred methods for the determination of antioxidant potential [37]. According to DPPH assay, nectarine (1.42 ± 0.04 mg AAE/g) and apricot presented similar antioxidant potential which were significantly higher (*p* ≤ 0.05) than peach and plum samples (Table 1). Our DPPH results are consistent with previous study conducted on Californian grown peach, plum and nectarine [20]. However, compared with another study on plum in North Pakistan, our DPPH value was slightly higher [48]. This variation could be explained by the different extraction solvent used, cultivars, growing region and climatic conditions. The previous study used water and acetone to extract phenolic compounds, while we used ethanol for extraction of phenolic compounds. It may suggest that ethanol could be a better solvent for phenolics extraction.

The FRAP activity of nectarine (0.98 ± 0.02 mg AAE/g) and apricot was higher as compared to other stone fruit samples. Compared with the *“Gönci magyarkajszi”* and *“Preventa”* apricot (1.76 AAE mg/ml) grown in Central Hungary, our stone fruits’ antioxidant potential was lower might be due to the difference of varieties and growing region [49]. In another study, FRAP of 27 different apricot cultivars ranged from 0.47 to 10.35 mmol AAE/L, which was also slightly lower than our result [50]. It has been emphasized that the variation of reducing capacity could be due to diverse regions, cultivars and harvest year and type of solvents used for extraction [50]. In addition, it was suggested that the FRAP activity was associated with types of phenolics and their composition. The extractable phenolic compounds showed higher FRAP values as compared to non-extractable phenolic compounds [51]. 

In ABTS, peach waste (0.43 ± 0.09 mg AAE/g) had the greatest radical scavenging capacity (*p* ≤ 0.05) compared to other stone fruit waste. Compared with a previous study, ABTS of 17 Luxembourgish grown plum cultivars range from 195 to 386 mg AAE/100 g, our data was slightly higher than Kaulmann, et al. [52] study. The variation is due to the extracted solvent of methanol, which was different from our 70% ethanolic extraction. However, another study focused on dry apricot fruit in Jammu showed higher ABTS [53]. In terms of TAC, nectarine waste (0.91 ± 0.09 mg AAE/g) presented the highest TAC value, followed by plum and apricot. The previous work about peach and apricot growing in Algeria showed lower antioxidant capacity as compared to our study [54]. The variation could be related to differences in varieties and growing region and type of solvent extraction. Another study on stone fruits of northern Greece showed slightly higher results of 15.13 ± 4.44 µmol AAE/g for plum, 14.16 ± 4.12 µmol AAE/g for peach, 10.40 ± 0.56 µmol AAE/g for nectarine and 4.00 ± 0.80 µmol AAE/g for apricot as compared to our study [55]. The variation could be due to difference in solute to solvent ratio, grown region and cultivars. Furthermore, Hui, et al. [51] argued that extractable phenolic compounds contribute more to total antioxidant capacity than non-extractable phenolic compounds. Combined with our data, nectarine waste may contain more extractable phenolic compounds than other stone fruits waste.

### 3.3. Correlation between Phenolic Compounds and Antioxidant Assays

Correlation analysis was applied to explain the relationship between TPC, TFC, TTC and antioxidant assays (DPPH, FRAP, ABTS and TAC), performed with Pearson’s correlation test (Table 2). The TPC was strongly positively correlated with TFC (r = 0.982, *p* ≤ 0.01), whereas DPPH is positively correlated with ABTS (r = 0.960, *p* ≤ 0.05). Since flavonoids are benzo-γ-pyrone derivatives composed of polyphenolic and pyrane rings [56], the strong and positive correlation between TPC and TFC could indicate that phenolic content in stone fruits waste are composed of a high concentration of flavonoids. The similar relationship between TPC and TFC was also confirmed in the previous study [41].

In terms of antioxidant assays, DPPH and ABTS were applied to determine the free radical scavenging capacity. The positive relationship between DPPH and ABTS has also been confirmed in previous study [41]. However, another antioxidant assay, FRAP, was not strongly correlated with DPPH and ABTS. Since these three assays measure the scavenging ability differently, the stability of radicals and the mechanisms can influence the result [8]. For instance, it has been reported that DPPH was applied to detect the hydrogen donator, while FRAP was based on electron transfer [57]. The difference between the result of FRAP and DPPH has also been found in the study of Dudonne, et al. [58]. 

However, the correlation between antioxidant activities and phenolic content was contradictory. Some authors observed that there were strong correlations between antioxidant activities and total phenolic content, whereas some represent low or no relationship [41,56,59]. Since we did not find a high correlation, it could be inferred that phenolic compounds are not the only bioactive compounds in stone fruits waste which contribute to antioxidant activity. In addition, although plum had higher TPC and TFC value, the antioxidant activity of plum was lower. Since all the antioxidant assays are not only aimed at estimating phenolic compounds but all types of phytochemicals and bioactive compounds, the antioxidant results may be influenced by other phytochemicals, for example, the carotenoids [60]. However, we could conclude that stone fruits waste contains many phenolics and has antioxidant potential. Therefore, we conducted LC-ESI-QTOF-MS/MS and HPLC-PDA to qualify and quantify phenolics present in stone fruits waste.

### 3.4. Phenolic Identification by LC-ESI-QTOF-MS/MS

LC-ESI-QTOF-MS/MS was applied to analyze the phenolic compounds from the stone fruits samples in negative and positive ionization modes. All the compounds identified in the stone fruits samples were based on the mass-to-charge (m/z) values of mass spectrometry in negative ionization and positive ionization modes (Supplementary data—Figures S1 and S2). The Agilent LC/MS MassHunter Qualitative Software and Personal Compound Database and Library (PCDL) with their online databases were applied to analyze the compounds. Among them, we selected compounds with a PCDL score more than 80 and a mass error <±5 ppm to conduct further characterization and verification.

As shown in Table 3, identified compounds were listed along with their molecular formula, retention times, ionization modes, molecular weight, theoretical weight, observed wight, mass error and MS/MS product ions. LC-ESI-QTOF-MS/MS has tentatively characterized 59 phenolic compounds in four stone fruits waste including 26 phenolic acids, 28 flavonoids, 1 lignans and 4 other polyphenols.

#### 3.4.1. MS/MS Based Characterization of Phenolic Compounds

##### Phenolic Cids

In terms of phenolic acids, four sub-classes have been found in stone fruit waste samples, which includes 16 hydroxycinnamic acids, 6 hydroxybenzoic acids, 3 hydroxyphenylpropanoic acids and 1 hydroxyphenylacetic acid.

Hydroxycinnamic acids

Hydroxycinnamic acids, which are commonly found in different fruits, such as peach, plum, blueberry and mango, have been reported to have antioxidant potential [61]. In this study, 16 hydroxycinnamic acids were detected, which showed the largest number than any other sub-classes.

The presence of 3-caffeoylquinic acid (Compound **6** with [M − H]^−^ m/z at 353.0864) was confirmed by the product ions of m/z 253 [M − H − HCOOH − 3H_2_O (loss of 100 Da), m/z 190 [M − H − C_6_H_5_O_2_ − 3H_2_O] (loss of 163 Da) and m/z 144 [M − H − C_7_H_11_O_6_ − H_2_O] (loss of 209 Da) from the parent ion [62]. A similar compound, 3-caffeoylquinic acid was previously found in Chinese peach and nectarine by UPLC-ESI-QTOF-MS analysis and was reported as natural antioxidant [63,64]. Ferulic acid (Compound **9**) was presented in nectarine, plum and apricot with [M − H]^−^ m/z at 193.0501 in the negative ionization mode. The identification of ferulic acid was achieved by the MS^2^ experiment which displayed the product ions at m/z 178, m/z 149 and m/z 134, indicating the loss of CH_3_, CO_2_ and CH_3_ with CO_2_ from the precursor, respectively [65]. Regarding to previous research, ferulic acid has been characterized in fresh Japanese plums by HPLC [66]. Furthermore, ferulic acid was reported to have the ability of free radical scavenging and inhibit the toxicity of free radicals [67].

Compounds **7, 11, 13** which were characterized to be 3-feruloylquinic acid, 3-*p*-coumaroylquinic acid and caffeic acid, respectively, were reported in different stone fruits in previous studies [29,66,68]. To our best knowledge, isoferulic acid 3-sulfate, cinnamic acid, caffeoyl glucose, 1-sinapoyl-2-feruloylgentiobiose and hydroxycaffeic acid (Compounds **2, 3, 4, 14, 15**) were identified first time in stone fruits; however, they were already reported in other plants. For example, isoferulic acid 3-sulfate and hydroxycaffeic acid have been found in berries in previous studies [69,70], Compound **3** was reported in peach leaves, sesame and almond [71,72]. Moreover, Chokanan mango was reported to be rich in caffeoyl glucose (Compound **4**) [73] and 1-Sinapoyl-2-feruloylgentiobiose (Compound **14**) has been found in various of cruciferous vegetables [74].

Hydroxybenzoic acids

Hydroxybenzoic acids were widely present in fruits and vegetables and were reported to have antioxidant activity and have the potential to ameliorate cardiovascular disorders [75]. 

In present work, six hydroxybenzoic acids were identified and tentatively characterized. Compound **20** ([M − H]^−^ m/z at 137.0240) and Compound **21** ([M − H]^−^ m/z at 153.0190) were tentatively characterized as 2-hydroxybenzoic acid and 2,3-dihydroxybenzoic acid based on the product ions at m/z 93 and at m/z 109, due to the loss of CO_2_ (44 Da) from the precursor ions [76,77]. In previous studies, these two phenolic acids have been reported as important functional compounds in peach [78,79]. Compound **17**, Compound **18** and Compound **22** were tentatively characterized to be ellagic acid acetyl-xyloside, gallic acid 4-*O*-glucoside and 3-*O*-methylgallic acid, respectively. To the best of our knowledge, these compounds were identified for the first time in stone fruits; however, they already reported in guava, raspberry and seaweed [80,81,82].

Hydroxyphenylpropanoic acids and hydroxyphenylacetic acids

According to our results, three hydroxyphenylpropanoic acids and one hydroxyphenylacetic acid were tentatively characterized in stone fruit waste samples.

Compound **23** (Dihydroferulic acid 4*-O-*glucuronide) was detected only in the negative ionization mode with the [M − H]^−^ precursor ions at m/z 371.0988. The characteristic loss of the glucuronide (176 Da) moiety was observed, which produced the product ions at m/z 195 [83]. Most of the hydroxyphenylpropanoic acids and hydroxyphenylacetic acids were detected for the first time in stone fruits waste. Two out of three hydroxyphenylpropanoic acid derivatives (Compound **24, 25**) were detected only in peach and have been reported in palm fruit [40]. Dihydroferulic acid 4*-O-*glucuronide (Compound **23**) was reported in *Opuntia ficus-indica* fruit with antioxidant potential by Aruwa, et al. [84]. Compound **26** was identified in all stone fruits and previously reported in different mango peel samples by Peng, et al. [37].

##### Flavonoids

Focusing on flavonoids, eight sub-classes have been identified in stone fruit samples, including eight flavonols, five flavanols, four flavones, three isoflavonoids, three flavanones, three dihydroflavonols, one anthocyanin and one dihydrochalcone.

Flavonols

Flavonols are common flavonoids and have been found to have antioxidant and antiatherogenic properties [85]. In this research, eight flavonols were tentatively characterized. Isorhamnetin (Compound **27**, [M − H]^−^ at m/z 315.0504) was found only in plum in negative mode, and identified according to the product ions at m/z 300 and m/z 271, corresponding to the loss of CH_3_ and CO_2_ from the precursor [86]. To our best knowledge, it is the first time to report this compound in stone fruits; however, it was previously found in citrus fruits [87]. Three kaempferol derivatives including Compound **30** (Kaempferol 3,7*-O-*diglucoside), Compound **32** (Kaempferol 3*-O-*glucosyl-rhamnosyl-galactoside) and Compound **33** (Kaempferol 3*-O-*(2″-rhamnosyl-galactoside) 7*-O-*rhamnoside) were tentatively characterized in our study. These derivatives has been reported previously in peach and other fruit samples [88]. Compound **30** was also detected in all stone fruit samples and previously reported in saffron [89].

Flavanols

Flavanols are reported in many fruits and vegetables with antioxidant and cardiovascular disease prevention properties [90]. In this study, five flavanols were tentatively identified in stone fruits waste.

Compound **35** and Compound **36** were identified as procyanidin dimer B1 and procyanidin trimer C1 appearing in most of the stone fruit waste samples based on the [M − H]^−^ m/z at 577.1342 and [M − H]^−^ m/z at 865.1959. The loss of 126 Da (phloroglucinol) from the precursor allowed the identification of procyanidin dimer B1 [91], while the identification of a procyanidin trimer C1 was achieved by comparing the MS^2^ with a previous study [92], which showed product ions at m/z 739, m/z 713 and m/z 695, representing the 126 Da loss of the heterocyclic ring fission (HRF) reaction, 152-Da loss of retro-Diels–Alder (RDA) and further loss of H_2_O. Procyanidin dimer B1 was found in peach, nectarine and plum as reported in the previous literature [63,66]. In contrast, procyanidin trimer C1 was first identified in stone fruits but it was previously found in mutamba fruit [93]. Compound (**37**), which appeared only in plum, was identified as Cinnamtannin A2. To the best of our knowledge, it was also found in stone fruits for the first time, but it has been reported previously in strawberry [92]. Compound (**38**) was detected as (+)-catechin in apricot, plum and peach samples. It has been confirmed that (+)-catechin is mostly found in stone fruits including plum, apricot, peach and cherry [68].

Flavones, isoflavonoids and flavanones

Flavones are components of various of edible plants, including fruits and vegetables and also present in beverages such as tea, wine and coffee. Because of their antioxidant, anti-microbial and anti-inflammatory activities, flavones could play an important role in metabolic diseases [94]. In terms of stone fruits, we tentatively characterized four flavones, three isoflavonoids and three flavanones. Compound **40** presenting only in peach in the negative mode was proposed as apigenin 7*-O-*(6″-malonyl-apiosyl-glucoside) based on the [M − H]^−^ m/z at 649.1429 and confirmed by the product ions at m/z 605, corresponding to the loss of CO_2_ (44 Da) from the precursor ion [95]. Compound **47** was tentatively characterized as neoeriocitrin based on the precursor ions [M − H]^−^ at m/z 595.1650. In the MS/MS experiment, neoeriocitrin was confirmed by product ions at m/z 431 [M − H − rhamnoside − H_2_O] and m/z 287 [M − H − rhamnoside − glucoside] [96]. To the best of our knowledge, most of these derivatives were detected for the first time in stone fruits waste. However, they could be found in other edible plants. 

Compound **43,** Apigenin 6-C-glucoside was detected in the plum sample, previous reported in *Bryonia dioica* and citrus fruits [97,98]. Another flavone (Compound **40**) which was found in the peach sample was previously reported in tomato by Lucini, et al. [99]. Two of the flavones (Compounds **41, 42**) were found in apricot, peach, and plum and were identified as apigenin 6,8-di-C-glucoside and 6-hydroxyluteolin 7-*O*-rhamnoside, respectively. Apigenin 6,8-di-C-glucoside was reported in tropical citrus fruits, while 6-hydroxyluteolin 7-*O*-rhamnoside were found in dry seed, including sesame and sunflower [100,101]. Compounds **44** and **45** were identified in peach and were previously detected in soy milk and roots of *Pongamia pinnata*, respectively [102,103]. Compound (**46**), found only in apricot, has been reported in pomegranate in a previous study [104]. As for flavanones, neoeriocitrin and narirutin were also detected in nectarine in our study. Previously, it has been reported that chinotto also contained neoeriocitrin [105], while narirutin was identified in citrus fruits [106].

Dihydroflavonols, dihydrochalcones and anthocyanins

Dihydroflavonols, dihydrochalcones and anthocyanins were proved to have free radical scavenging capacity [107]. In our study, three dihydroflavonols, one dihydrochalcone and one anthocyanin have been identified in stone fruits.

Dihydroquercetin (Compound **50**) and dihydromyricetin 3*-O-*rhamnoside (Compound **52**) and were detected in negative mode with [M − H]^−^ m/z at 303.0507 and m/z 465.1050. The identity of dihydroquercetin was confirmed by the fragment ions at m/z 285, m/z 275 and m/z 151, corresponding to the loss of H_2_O, CO and 152 Da loss by RDA cleavage [108], while dihydromyricetin 3*-O-*rhamnoside was confirmed by the product ion at m/z 301 [M − H − rhamnose, loss of 164 Da] [109]. Two out of three dihydroflavonols (Compounds **50** and **54**) were identified in the peach sample and these compounds were already reported in different peach varieties [110]. Dihydroquercetin 3-*O*-rhamnoside (Compound **52**) was found only in nectarine. Previously, it has also been reported in grape skins [111]. Dihydromyricetin 3-*O*-rhamnoside (Compound **51**) which was detected in nectarine, peach and plum has been reported in the khat plant which grew in Ethiopia [112].

##### Lignans

Lignans are bioactive compounds with anti-inflammatory, anti-oxidant and anti-tumor activities [113]. Only one lignan was tentatively characterized in our study. Compound **55** was identified as 7-hydroxymatairesinol according to the [M − H]^−^ at m/z 373.1298 in nectarine. As per our best knowledge, 7-hydroxymatairesinol was reported first time in stone fruits; however, it was found in various seeds, including sunflower and pumpkin [101].

##### Other polyphenols

As for other polyphenols, three sub-classes in stone fruits waste have been characterized, which include two hydroxybenzaldehydes, one hydroxycoumarin and one tyrosol.

Compound **56** appeared both in peach and plum and was tentatively characterized as 4-hydroxybenzaldehyde based on the precursor ion at [M − H]^−^ at m/z 121.0298 and confirmed based on the MS^2^ fragmentation, which exhibited the loss of CO_2_ from the precursor, resulting in the product ion at m/z 77 [114]. It has been reported in sweet cherry, which is another stone fruit [115]. To the best of our knowledge, *p*-anisaldehyde and scopoletin (Compounds **57** and **58**) which were presented in different stone fruits, were first identified in stone fruits while they were previously found in hawthorn [116]. 3,4-DHPEA-AC (Compound **59**), which belongs to tyrosol derivatives, has also been found in olive oil [117].

The results of LC-ESI-QTOF-MS/MS illustrated that phenolic compounds are important components of stone fruits, especially hydroxycinnamic acid derivatives, hydroxybenzoic acid derivatives, flavonols, flavanols and other polyphenols. These phenolic compounds have significant free radical scavenging capacity and antioxidant activity. Hence, stone fruits waste may have antioxidant potential and could be used for functional foods, nutraceuticals and pharmaceuticals.

#### 3.4.2. Distribution of Phenolic Compounds—Venn Diagram

**Figure 1:**
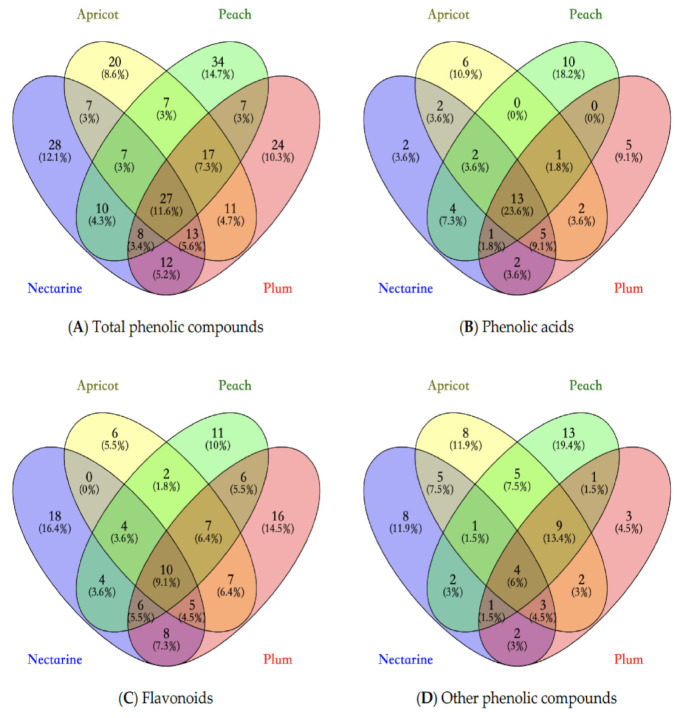
Venn diagram of phenolic compounds presented in stone fruits waste samples. (**A**) shows the relations of total phenolic compounds present in stone fruits waste; (**B**) shows the relations of phenolic acids in present in stone fruits waste; (**C**) shows the relations of flavonoids present in stone fruits waste; (**D**) shows the relations of other phenolic compounds present in stone fruits waste.

As shown in Figure 1, Venn diagrams were applied to indicate the distribution of the phenolic compounds in four stone fruits’ waste. The comparison illustrated that there were differences between the composition of phenolic compounds in these four stone fruits.

Based on Figure 1A, a total of 232 phenolic compounds were identified in four stone fruits waste samples. Among them, 11.6% phenolic compounds were detected in all samples. According to Figure 1B–D, 23.6% phenolic acids, 9.1% flavonoids and 6% other phenolic compounds were found in all four samples, respectively. The proportion of common characterized compounds of flavonoids in all stone fruits samples was similar to that of the total phenolic compounds. This could indicate that, although the peach, nectarine, plum and apricot are different fruits, their compositions of flavonoids are likely to be similar. However, the proportions of shared compounds of phenolic acids and other phenolic compounds were different from that of total phenolic compounds. It could be argued that phenolic acids and other phenolic compounds contributed more to the differences in phenolic compounds and antioxidant activities among these stone fruits. In addition, by comparing the shared compounds between any two stone fruits samples, the phenolic composition of peach and nectarine were similar. However, the composition of apricot was different from that of peach, nectarine and plum. Previous study has also reported that the composition of antioxidants in peach and apricot were significantly different [118]. Furthermore, it has been found that genotype, pre-harvest, post-harvest and climatic conditions could influence the phenolic profile of stone fruits [119,120].

By conducting the Venn diagram, we could argue that the composition of phenolic compounds is different among four stone fruits. However, the influence of specific phenolic compounds and related bioactivities should be investigated in future research.

### 3.5. HPLC-PDA Analysis

HPLC is commonly used to determine the quantification of phenolic compounds present in different fruits and vegetables [121]. The targeted phenolic compounds are detected by the UV spectra and quantified by the retention times. In this research, the HPLC-PDA was applied to quantify the phenolic composition of stone fruits waste. In our research, 10 targeted phenolic compounds, including five phenolic acids and five flavonoids, were quantified. As shown in Table 4, *p*-hydroxybenzoic acid was the highest phenolic acid and quercetin is the most dominant flavonoid.

According to Table 4, peach and plum showed significantly higher concentrations of phenolic acids which support our TPC results. *p*-Hydroxybenzoic acid was the most dominant phenolic acid in peach (18.64 ± 1.30 mg/g), nectarine and plum, respectively. However, in apricot, chlorogenic acid exhibited the highest value. Plum waste contained all five targeted phenolic acids with the lowest concentration in gallic acid. In a previous study, different cultivars of peaches and nectarines grown in southern Serbia were extracted by 80% acetone (v/v) and the phenolic profile was determined by HPLC-photodiode array detection (DAD), their results illustrated that some phenolic acids, for example chlorogenic acid in *“Vesna”* peach (126.1 mg/kg) was higher than that in *“Fantasia”* nectarine (31.3 mg/kg). Our phenolic concentration was slightly higher than previous published studies, which may be explained by a different cultivar, growing regions and different extraction solvents and methods applied [122]. Previously, Biesaga, et al. [123] have also quantified the concentration of chlorogenic acid, gallic acid and *p*-hydroxybenzoic acid from Polish grown plums at different ripening stages and reported that the concentration of phenolic acids decrease during ripening.

In terms of flavonoids, peach and plum also contain higher concentration than others, which confirms the TFC result. Quercetin was the most dominant flavonoid in peach (19.68 ± 1.38 mg/g), nectarine and plum while catechin is the highest flavonoid in apricot. Moreover, peach contained more flavonoids than nectarine was in agreement of the previous study [122]. Preciously, Campbell and Padilla-Zakour [118] conducted HPLC to determine several flavonoids in canned American grown peaches and apricots which were extracted by methanol/water solution. However, the concentration of flavonoids in *“Harogem”* apricot were higher than that in *“Redhaven”* peach, which is different to our research. Regarding the catechin and epicatechin, the concentration of catechin was higher than epicatechin in all four Australian grown stone fruits waste. Previously, Liao, et al. [124] quantified the concentration of catechin and epicatechin in Georgian grown peach cultivars, including “*Carored*”, “*Golden Prince*”, “*Ruby Prince*”, “*August Prince*” and “*O’Henry*” and confirmed that catechin and epicatechin in peach were higher than nectarine. The difference in results could be due to different extraction method and type of solvents and samples [125].

## 4. Conclusions

Based on this research, it is concluded that plum waste contained higher concentrations of total phenolic compounds and flavonoids than other stone fruit wastes, while apricot had higher concentration of tannins. Moreover, nectarine had higher antioxidant capacity in DPPH, FRAP and TAC assays as compared to other stone fruit waste. The LC-ESI-QTOF-MS/MS showed phenolic profiling in stone fruit waste while HPLC-PDA confirmed that targeted phenolic compounds were significantly higher in plum and peach as compared to other stone fruit waste. In short, the obtained results could support the applications and repurposing of stone fruits waste for functional foods, feed, nutraceuticals and pharmaceutical industries.

## Figures and Tables

**Table 1:** Phenolic content and antioxidant activities in stone fruit samples.

Antioxidant Assays	Peach	Nectarine	Plum	Apricot
TPC (mg GAE/g)	0.47 ± 0.08 ^c^	0.31 ± 0.05 ^d^	0.94 ± 0.07 ^a^	0.65 ± 0.12 ^b^
TFC (mg QE/g)	0.18 ± 0.01 ^c^	0.16 ± 0.09 ^c^	0.34 ± 0.01 ^a^	0.23 ± 0.07 ^b^
TTC (mg CE/g)	0.07 ± 0.02 ^c^	0.10 ± 0.06 ^b^	0.09 ± 0.02 ^c^	0.19 ± 0.03 ^a^
DPPH (mg AAE/g)	0.98 ± 0.07 ^b^	1.42 ± 0.04 ^a^	0.94 ± 0.17 ^b^	1.47 ± 0.12 ^a^
FRAP (mg AAE/g)	0.54 ± 0.01 ^c^	0.98 ± 0.02 ^a^	0.63 ± 0.04 ^b^	0.93 ± 0.04 ^a^
ABTS (mg AAE/g)	0.43 ± 0.09 ^a^	0.23 ± 0.04 ^b^	0.21 ± 0.01 ^b^	0.25 ± 0.11 ^b^
TAC (mg AAE/g)	0.27 ± 0.10 ^d^	0.91 ± 0.09 ^a^	0.61 ± 0.12 ^b^	0.54 ± 0.09 ^c^

All data are the mean ± standard deviation of three replicates. Means followed by different letters (^a, b, c, d^) within the same row are significantly different (*p* ≤ 0.05) from each other by using one-way analysis of variance and Tukey’s test. Data of stone fruit samples are reported on a fresh weight basis. GAE, gallic acid equivalents; QE, quercetin equivalents; CE, catechin equivalents; AAE, ascorbic acid equivalents.

**Table 2:** Pearson’s correlation coefficients (r) for the relationships between antioxidant assays and phenolic content.

Variables	TPC	TFC	TTC	DPPH	FRAP	ABTS
**TFC**	0.982 **					
**TTC**	0.124	0.040				
**DPPH**	−0.482	−0.485	0.744			
**ABTS**	−0.400	−0.353	0.653	0.960 *		
**FRAP**	−0.369	−0.477	−0.402	−0.396	−0.604	
**TAC**	−0.221	−0.067	0.125	0.550	0.756	−0.799

** Significant correlation with *p* ≤ 0.01; * Significant correlation with *p* ≤ 0.05.

**Table 3:** Qualitative characterization of phenolic compounds in stone fruits waste by Liquid chromatography coupled with electrospray-ionization quadrupole time-of-flight mass spectrometry (LC-ESI-QTOF-MS/MS).

No.	Compound Name	Moleular Formula	RT (min)	Ionization (ESI^−^/ESI^+^)	Molecular Weight	Theoretical Weight (m/z)	Observed Weight (m/z)	Error (ppm)	MS/MS Product Ions	Samples
**Phenolic acids**										
	**Hydroxycinnamic acids**									
1	1,5-Dicaffeoylquinic acid	C_25_H_24_O_12_	4.134	[M − H]^−^	516.1268	515.1195	515.1198	0.6	353, 335, 191, 179	NE
2	Isoferulic acid 3-sulfate	C_10_H_10_O_7_S	5.341	[M − H]^−^	274.0147	273.0074	273.0067	−2.6	193, 178	PL
3	Cinnamic acid	C_9_H_8_O_2_	9.317	[M − H]^−^	148.0524	147.0451	147.0449	−1.4	103	NE, *AP, PL
4	Caffeoyl glucose	C_15_H_18_O_9_	14.833	[M − H]^−^	342.0951	341.0878	341.0887	2.6	179, 161	NE, *PL
5	*p*-Coumaric acid 4-*O*-glucoside	C_15_H_18_O_8_	14.953	[M − H]^−^	326.1002	325.0929	325.0926	−0.9	163	*PE, PL
6	3-Caffeoylquinic acid	C_16_H_18_O_9_	20.038	[M − H]^−^	354.0951	353.0878	353.0864	−4.0	253, 190, 144	*NE, AP, PE, PL
7	3-Feruloylquinic acid	C_17_H_20_O_9_	20.817	[M − H]^−^	368.1107	367.1034	367.1023	−3.0	298, 288, 192, 191	NE, AP, *PE, PL
8	Ferulic acid 4-*O*-glucuronide	C_16_H_18_O_10_	22.305	[M − H]^−^	370.0900	369.0827	369.0826	−0.3	193	AP, *PL
9	Ferulic acid	C_10_H_10_O_4_	23.467	[M − H]^−^	194.0579	193.0506	193.0501	−2.6	178, 149, 134	*NE, PL, AP
10	Ferulic acid 4-*O*-glucoside	C_16_H_20_O_9_	23.500	[M − H]^−^	356.1107	355.1034	355.1032	−0.6	193, 178, 149, 134	*NE, AP, PL
11	3-*p*-Coumaroylquinic acid	C_16_H_18_O_8_	27.013	[M − H]^−^	338.1002	337.0929	337.0918	−3.3	265, 173, 162	PL, *PE, AP, NE
12	*m*-Coumaric acid	C_9_H_8_O_3_	27.808	[M − H]^−^	164.0473	163.0400	163.0394	−3.7	119	*NE, AP, PE, PL
13	Caffeic acid	C_9_H_8_O_4_	32.032	[M − H]^−^	180.0423	179.0350	179.0347	−1.7	143, 133	*NE, PL
14	1-Sinapoyl-2-feruloylgentiobiose	C_33_H_40_O_18_	36.370	[M − H]^−^	724.2215	723.2142	723.2124	−2.5	529, 499	AP
15	Hydroxycaffeic acid	C_9_H_8_O_5_	37.033	[M − H]^−^	196.0372	195.0299	195.0298	−0.5	151	PE, *PL
16	3-Sinapoylquinic acid	C_18_H_22_O_10_	41.574	[M − H]^−^	398.1213	397.1140	397.1129	−2.8	233, 179	NE
	**Hydroxybenzoic acids**									
17	Ellagic acid acetyl-xyloside	C_21_H_16_O_13_	4.101	[M − H]^−^	476.0591	475.0518	475.0498	−4.2	301	PE
18	Gallic acid 4-*O*-glucoside	C_13_H_16_O_10_	6.914	[M − H]^−^	332.0743	331.0670	331.0675	1.5	169, 125	*PL, AP
19	Protocatechuic acid 4-*O*-glucoside	C_13_H_16_O_9_	7.382	[M − H]^−^	316.0794	315.0721	315.0732	3.5	153	*PE, APt, PL
20	2-Hydroxybenzoic acid	C_7_H_6_O_3_	20.237	[M − H]^−^	138.0317	137.0244	137.0240	−2.9	93	*NE, AP, PE
21	2,3-Dihydroxybenzoic acid	C_7_H_6_O_4_	32.082	[M − H]^−^	154.0266	153.0193	153.0190	−2.0	109	*NE, PE, PL
22	3-*O*-Methylgallic acid	C_8_H_8_O_5_	84.390	**[M + H]^+^	184.0372	185.0445	185.0450	2.7	170, 142	PE
	**Hydroxyphenylpropanoic acids**									
23	Dihydroferulic acid 4-*O*-glucuronide	C_16_H_20_O_10_	5.689	[M − H]^−^	372.1056	371.0983	371.0988	1.3	195	NE, AP, *PL
24	3-Hydroxy-3-(3-hydroxyphenyl)propionic acid	C_9_H_10_O_4_	7.083	[M − H]^−^	182.0579	181.0506	181.0511	2.8	163, 135, 119	PE
25	Dihydrocaffeic acid 3-*O*-glucuronide	C_15_H_18_O_10_	17.454	[M − H]^−^	358.0900	357.0827	357.0819	−2.2	181	PE
	**Hydroxyphenylacetic acids**									
26	3,4-Dihydroxyphenylacetic acid	C_8_H_8_O_4_	20.715	[M − H]^−^	168.0423	167.0350	167.0344	−3.6	149, 123	NE, *AP, PE, PL
**Flavonoids**										
	**Flavonols**									
27	Isorhamnetin	C_16_H_12_O_7_	27.076	[M − H]^−^	316.0583	315.0510	315.0504	−1.9	300, 271	PL
28	Myricetin 3-*O*-rutinoside	C_27_H_30_O_17_	32.960	[M − H]^−^	626.1483	625.1410	625.1399	−1.8	301	*NE, PE
29	Quercetin 3-*O*-glucosyl-xyloside	C_26_H_28_O_16_	34.730	[M − H]^−^	596.1377	595.1304	595.1290	−2.4	265, 138, 116	NE, *PL
30	Kaempferol 3,7-*O*-diglucoside	C_27_H_30_O_16_	37.284	[M − H]^−^	610.1534	609.1461	609.1445	−2.6	447, 285	*NE, AP, PE, PL
31	Myricetin 3-*O*-rhamnoside	C_21_H_20_O_12_	39.355	[M − H]^−^	464.0955	463.0882	463.0874	−1.7	317	*NE, PE, PL
32	Kaempferol 3-*O*-glucosyl-rhamnosyl-galactoside	C_33_H_40_O_20_	40.283	[M − H]^−^	756.2113	755.2040	755.2064	3.2	285	*PE, PL
33	Kaempferol 3-*O-*(2″-rhamnosyl-galactoside) 7-*O*-rhamnoside	C_33_H_40_O_19_	42.036	[M − H]^−^	740.2164	739.2091	739.2106	2.0	593, 447, 285	AP, *PL
34	Quercetin 3-*O-*arabinoside	C_20_H_18_O_11_	42.798	[M − H]^−^	434.0849	433.0776	433.0772	−0.9	301	PE, *PL
	**Flavanols**									
35	Procyanidin dimer B1	C_30_H_26_O_12_	17.139	[M − H]^−^	578.1424	577.1351	577.1342	−1.6	451	NE, *PE, PL
36	Procyanidin trimer C1	C_45_H_38_O_18_	19.177	[M − H]^−^	866.2058	865.1985	865.1959	−3.0	739, 713, 695	*PE, PL
37	Cinnamtannin A2	C_60_H_50_O_24_	19.422	[M − H]^−^	1154.2692	1153.2619	1153.2609	−0.9	739	PL
38	(+)-Catechin	C_15_H_14_O_6_	19.704	[M − H]^−^	290.0790	289.0717	289.0717	0.0	245, 205, 179	*AP, PL, PE
39	4′-*O*-Methyl-(-)-epigallocatechin 7-*O*-glucuronide	C_22_H_24_O_13_	32.112	[M − H]^−^	496.1217	495.1144	495.1138	−1.2	451, 313	NE, PE, *PL
	**Flavones**									
40	Apigenin 7-*O*-(6″-malonyl-apiosyl-glucoside)	C_29_H_30_O_17_	4.416	[M − H]^−^	650.1483	649.1410	649.1429	2.9	605	PE
41	Apigenin 6,8-di-C-glucoside	C_27_H_30_O_15_	43.461	[M − H]^−^	594.1585	593.1512	593.1500	−2.0	503, 473	*AP, PE, PL
42	6-Hydroxyluteolin 7-*O*-rhamnoside	C_21_H_20_O_11_	46.460	[M − H]^−^	448.1006	447.0933	447.0938	1.1	301	*AP, PE, PL
43	Apigenin 6-C-glucoside	C_21_H_20_O_10_	55.256	[M − H]^−^	432.1056	431.0983	431.0984	0.2	413, 341, 311	PL
	**Isoflavonoids**									
44	6″-*O*-Acetyldaidzin	C_23_H_22_O_10_	4.413	[M − H]^−^	458.1213	457.1140	457.1125	−3.3	221	PL
45	Violanone	C_17_H_16_O_6_	20.267	[M − H]^−^	316.0947	315.0874	315.0868	−1.9	300, 285, 135	PL
46	3′-Hydroxydaidzein	C_15_H_10_O_5_	81.970	[M + H]^+^	270.0528	271.0601	271.0610	3.3	253, 241, 225	AP
	**Flavanones**									
47	Neoeriocitrin	C_27_H_32_O_15_	34.931	[M − H]^−^	596.1741	595.1668	595.1650	−3.0	431, 287	NE
48	Narirutin	C_27_H_32_O_14_	41.624	[M − H]^−^	580.1792	579.1719	579.1696	−4.0	271	NE
49	Hesperetin 3′-*O*-glucuronide	C_22_H_22_O_12_	46.562	[M − H]^−^	478.1111	477.1038	477.1044	1.3	301, 175, 113, 85	NE, *PE
	**Dihydroflavonols**									
50	Dihydroquercetin	C_15_H_12_O_7_	5.775	[M − H]^−^	304.0583	303.0510	303.0507	−1.0	285, 275, 151	PE
51	Dihydromyricetin 3-*O*-rhamnoside	C_21_H_22_O_12_	23.846	[M − H]^−^	466.1111	465.1038	465.1050	2.6	301	*NE, PE, PL
52	Dihydroquercetin 3-*O*-rhamnoside	C_21_H_22_O_11_	27.029	[M − H]^−^	450.1162	449.1089	449.1069	−4.5	303	NE
	**Anthocyanins**									
53	Cyanidin 3-*O*-(2-*O*-(6-*O*-(E)-caffeoyl-D glucoside)-D-glucoside)-5-*O*-D-glucoside	C_43_H_49_O_24_	39.482	[M + H]^+^	949.2614	950.2687	950.2676	−1.2	787, 463, 301	PE
	**Dihydrochalcones**									
54	Phloridzin	C_21_H_24_O_10_	51.681	[M − H]^−^	436.1369	435.1296	435.1307	2.5	273	PE
**Lignans**										
55	7-Hydroxymatairesinol	C_20_H_22_O_7_	41.309	[M − H]^−^	374.1366	373.1293	373.1298	1.3	343, 313, 298, 285	NE
**Other polyphenols**										
	**Hydroxybenzaldehydes**									
56	4-Hydroxybenzaldehyde	C_7_H_6_O_2_	44.769	[M − H]^−^	122.0368	121.0295	121.0298	2.5	77	PE, *PL
57	*p*-Anisaldehyde	C_8_H_8_O_2_	55.681	**[M + H]^+^	136.0524	137.0597	137.0599	1.5	122, 109	NE, AP, PE, *PL
	**Hydroxycoumarins**									
58	Scopoletin	C_10_H_8_O_4_	36.851	[M − H]^−^	192.0423	191.0350	191.0345	−2.6	176	AP
	**Tyrosols**									
59	3,4-DHPEA-AC	C_10_H_12_O_4_	11.802	[M − H]^−^	196.0736	195.0663	195.0657	−3.1	135	AP

* Compound was detected in more than one stone fruit samples, data presented in this table are from asterisk sample. ** Compounds were detected in both negative [M − H]^−^ and positive [M + H]^+^ mode of ionization while only single mode data are presented. RT refers to retention time. Stone fruits samples mentioned in abbreviations are Peach “PE”, Plum “PL”, Apricot “AP’’, and Nectarine “NE”.

**Table 4:** Quantification of phenolic compounds in stone fruits waste samples by HPLC-photodiode array detection (PDA).

No.	Compound Name	Peach (mg/g fw)	Nectarine (mg/g fw)	Plum (mg/g fw)	Apricot (mg/g fw)	Phenolic Class
1	Gallic acid	2.98 ± 0.18 ^a^	-	2.75 ± 0.22 ^a^	1.25 ± 0.06 ^b^	Phenolic acids
2	Protocatechuic acid	-	1.27 ± 0.09 ^c^	3.12 ± 0.15 ^a^	2.47 ± 0.22 ^b^	Phenolic acids
3	*p*-Hydroxybenzoic acid	18.64 ± 1.30 ^a^	9.67 ± 0.48 ^b^	14.25 ± 0.99 ^a^	3.69 ± 0.26 ^c^	Phenolic acids
4	Chlorogenic acid	15.96 ± 0.80 ^a^	3.59 ± 0.32 ^c^	12.35 ± 0.74 ^a^	4.58 ± 0.23 ^b^	Phenolic acids
5	Caffeic acid	0.98 ± 0.06 ^c^	4.58 ± 0.27 ^a^	3.29 ± 0.26 ^b^	-	Phenolic acids
6	Catechin	7.45 ± 0.59 ^c^	9.64 ± 0.58 ^b^	14.58 ± 1.31 ^a^	7.58 ± 0.61 ^c^	Flavonoids
7	Epicatechin	1.25 ± 0.08 ^b^	0.78 ± 0.04 ^c^	2.39 ± 0.19 ^a^	1.39 ± 0.11 ^b^	Flavonoids
8	Epicatechin gallate	2.45 ± 0.12 ^a^	-	-	0.98 ± 0.06 ^b^	Flavonoids
9	Quercetin	19.68 ± 1.38 ^a^	12.47 ± 0.87 ^b^	14.78 ± 1.18 ^b^	6.37 ± 0.45 ^c^	Flavonoids
10	Kaempferol	5.98 ± 0.36 ^b^	2.17 ± 0.20 ^c^	7.98 ± 0.40 ^a^	1.87 ± 0.16 ^c^	Flavonoids

All data are the mean ± SD of three replicates. Means followed by different letters (^a, b, c^) within the same column are significantly different (*p* < 0.05) from each other. Data of peach, nectarine, plum and apricot waste are reported on a fresh weight basis.

## Data Availability

The data presented in this study are available in the supplementary material.

## Supplementary Materials

The following are available online at https://www.mdpi.com/2076-3921/10/2/234/s1. Figure S1: LC-ESI-QTOF-MS/MS basic peak chromatograph (BPC) for characterization of phenolic compounds of stone fruits waste. Figure S2: Extracted ion chromatogram of stone fruits waste samples and their mass spectra.
