# Supplementary material for: High-Throughput Screening and Characterization of Phenolic Compounds in Stone Fruits Waste by LC-ESI-QTOF-MS/MS and Their Potential Antioxidant Activities
Source: Antioxidants (Basel). 2021 Feb 4;10(2):234. doi: 10.3390/antiox10020234 (PMC7914583; doi:10.3390/antiox10020234)
Supplement: Supplementary file 1 [file antioxidants-10-00234-s001.pdf]

antioxidants

Supplementary data

High-Throughput Screening and Characterization of Phenolic Compounds in Stone Fruits Waste by LC-ESI-QTOF-MS/MS and their Potential Antioxidant Activities

Yili Hong [1], Zening Wang [1], Colin J. Barrow [2], Frank R. Dunshea [1,3] and Hafiz A.R. Suleria [1,2,*]

[1] School of Agriculture and Food, Faculty of Veterinary and Agricultural Sciences, The University of Melbourne, Parkville, VIC 3010, Australia; yilhong@student.unimelb.edu.au (Y.H.); zeningw1@student.unimelb.edu.au (Z.W.); fdunshea@unimelb.edu.au (F.R.D)
[2] Centre for Chemistry and Biotechnology, School of Life and Environmental Sciences, Deakin University, Waurn Ponds, VIC 3217, Australia; colin.barrow@deakin.edu.au (C.J.B)
[3] Faculty of Biological Sciences, The University of Leeds, Leeds LS2 9JT, UK
* Correspondence: hafiz.suleria@unimelb.edu.au; Tel.: +61-470-439-670

Abstract: Stone fruits, including peach (*Prunus persica* L.), nectarine (*Prunus nucipersica* L.), plum (*Prunus domestica* L.) and apricot (*Prunus armeniaca* L.) are common commercial fruits in the market. However, a huge amount of stone fruits waste is produced throughout the food supply chain during picking, handling, processing, packaging, storage, transportation, retailing and final consumption. These stone fruits waste contain high phenolic content which are the main contributors to the antioxidant potential and associated health benefits. The antioxidant results showed that plum waste contained higher concentrations of total phenolic content (TPC) (0.94 ± 0.07 mg gallic acid equivalents (GAE)/g) and total flavonoid content (TFC) (0.34 ± 0.01 mg quercetin equivalents (QE)/g), while apricot waste contained a higher concentration of total tannin content (TTC) (0.19 ± 0.03 mg catechin equivalents (CE)/g) and DPPH activity (1.47 ± 0.12 mg ascorbic acid equivalents (AAE)/g). However, nectarine waste had higher antioxidant capacity in ferric reducing-antioxidant power (FRAP) (0.98 ± 0.02 mg AAE/g) and total antioxidant capacity (TAC) (0.91 ± 0.09 mg AAE/g) assays, while peach waste showed higher antioxidant capacity in 2,2′-azino-bis-(3-ethylbenzothiazoline-6-sulfonic acid (ABTS) assay (0.43 ± 0.09 mg AAE/g) as compared to other stone fruits waste. Qualitative and quantitative phenolic analysis of Australian grown stone fruits waste were conducted by liquid chromatography coupled with electrospray-ionization quadrupole time-of-flight mass spectrometry (LC-ESI-QTOF-MS/MS) and HPLC-photodiode array detection (PDA). The LC-ESI-QTOF-MS/MS result indicates that 59 phenolic compounds were tentatively characterized in peach (33 compounds), nectarine (28), plum (38) and apricot (23). The HPLC-PDA indicated that *p*-hydroxybenzoic acid (18.64 ± 1.30 mg/g) was detected to be the most dominant phenolic acid and quercetin (19.68 ± 1.38 mg/g) was the most significant flavonoid in stone fruits waste. Hence, it could be concluded that stone fruit waste contains various phenolic compounds and have antioxidant potential. The results could support the applications of these stone fruit wastes in other food, feed, nutraceutical and pharmaceutical industries.

Keywords: Fruit waste; stone fruits; phenolic compounds; LC-ESI-QTOF-MS/MS; HPLC-PDA

Citation: Hong, Y.; Wang, Z.; Barrow, C.J.; Dunshea, F.R.; Hafiz A.R. Suleri High-Throughput Screening and Characterization of Phenolic Compounds in Stone Fruits Waste by LC-ESI-QTOF-MS/MS and their Potential Antioxidant Activities. *Antioxidants* **2021**, *10*, 234. https:// doi.org/10.3390/antiox 10020234

Academic Editor: Stefania D'Angelo
Received: 29 December 2020
Accepted: 26 January 2021
Published: 4 February 2021

Publisher's Note: MDPI stays neutral with regard to jurisdictional claims in published maps and institutional affiliations.

Copyright: © 2021 by the authors. Submitted for possible open access publication under the terms and conditions of the Creative Commons Attribution (CC BY) license (http://creativecommons.org/licenses/by/4.0/).

(a)

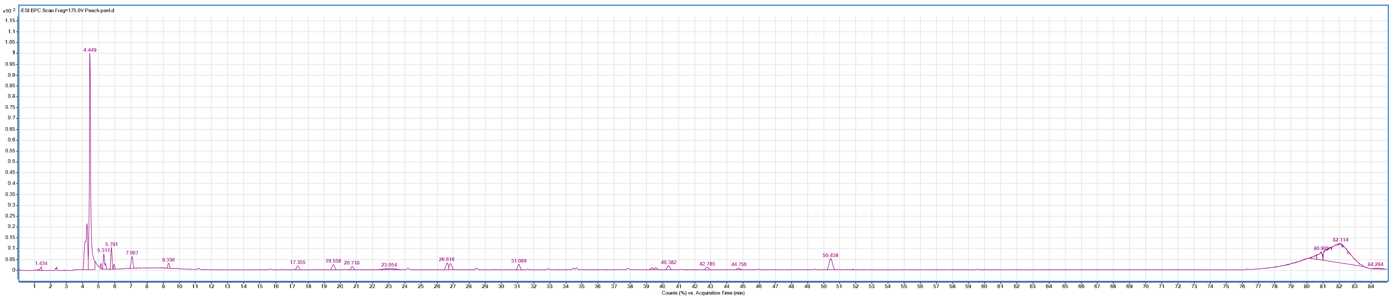

(b)

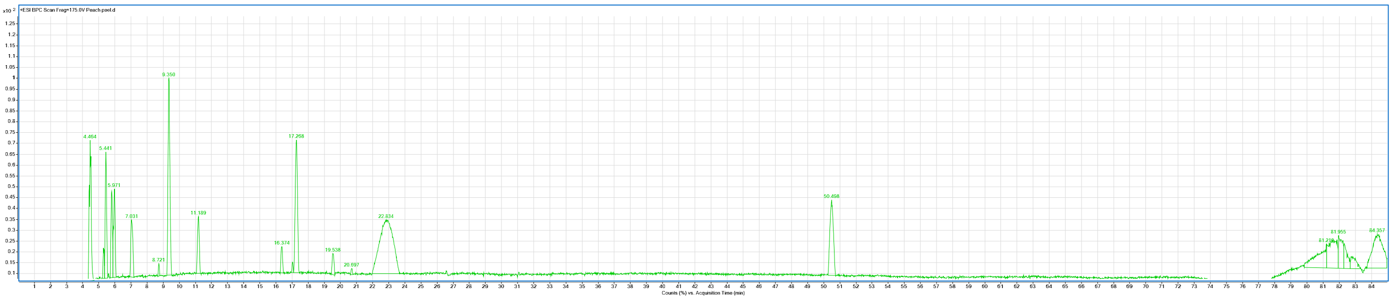

(c)

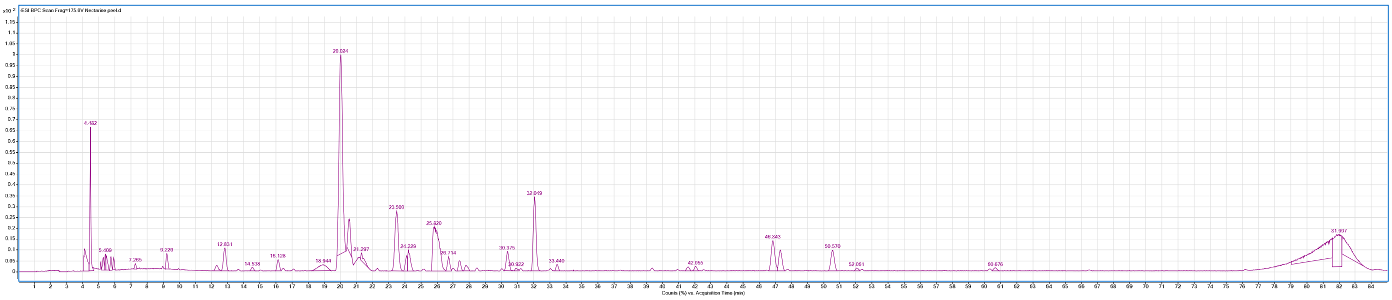

(d)

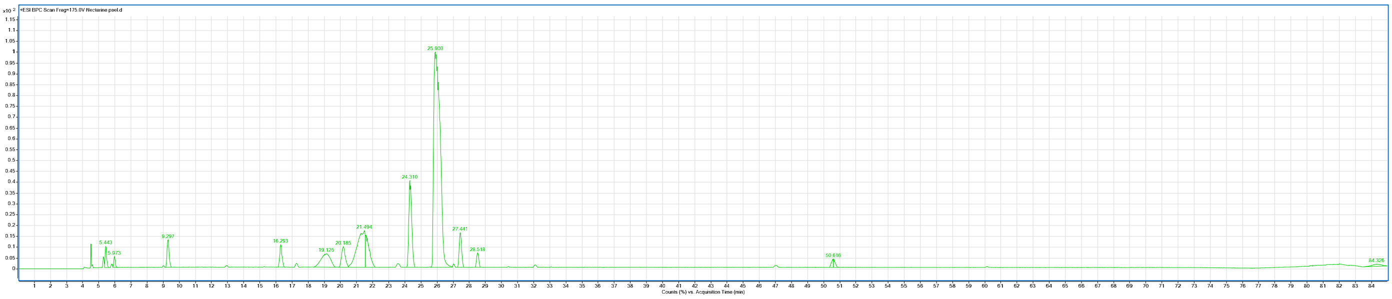

(e)

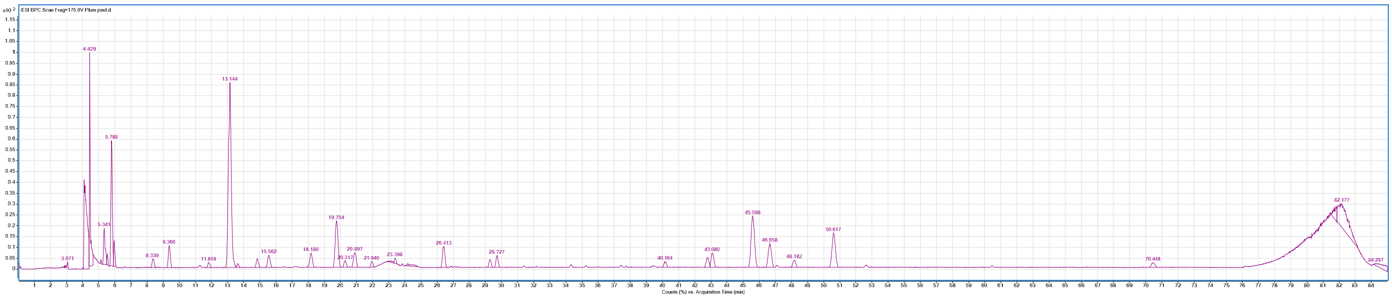

(f)

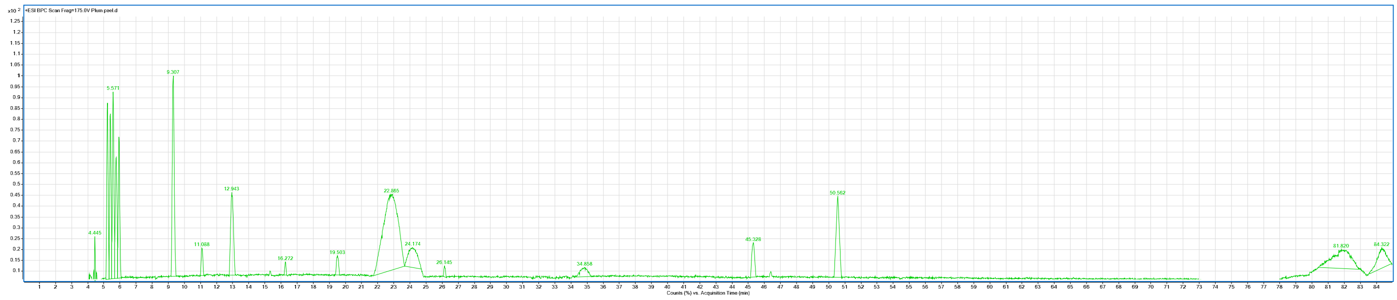

(g)

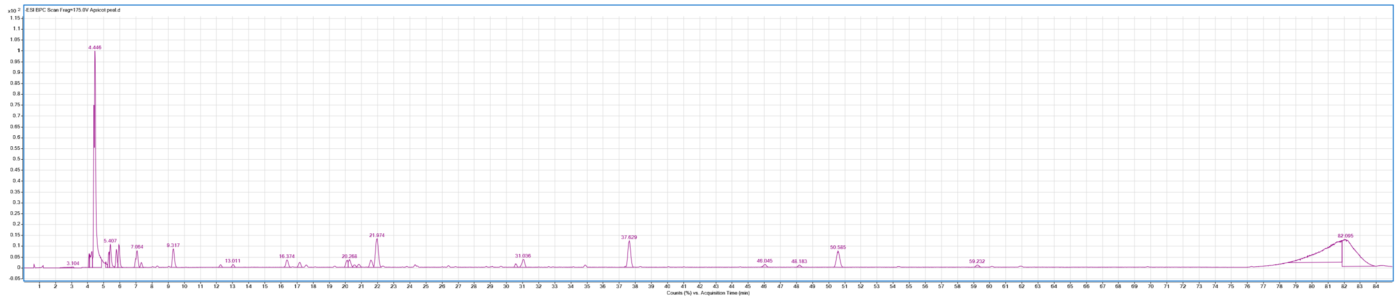

(h)

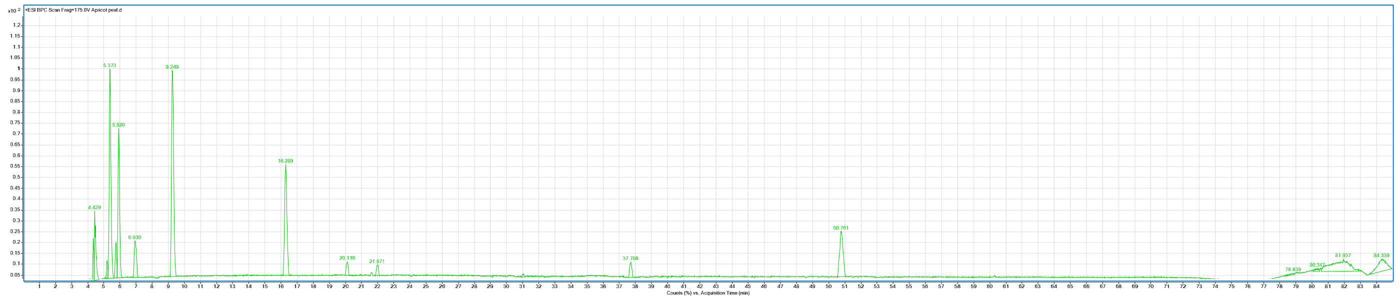

Figure 1S1. LC-ESI-QTOF-MS/MS basic peak chromatograph (BPC) for characterization of phenolic compounds of stone fruit waste samples; (a) Peach in negative ionization mode; **(b)** Peach in positive ionization mode; **(c)** Nectarine in negative ionization mode; **(d)** Nectarine in positive ionization mode; **(e)** Plum in negative ionization mode; **(f)** Plum in positive ionization mode; **(g)** Apricot in negative ionization mode; **(h)** Apricot in positive ionization mode.

(a)

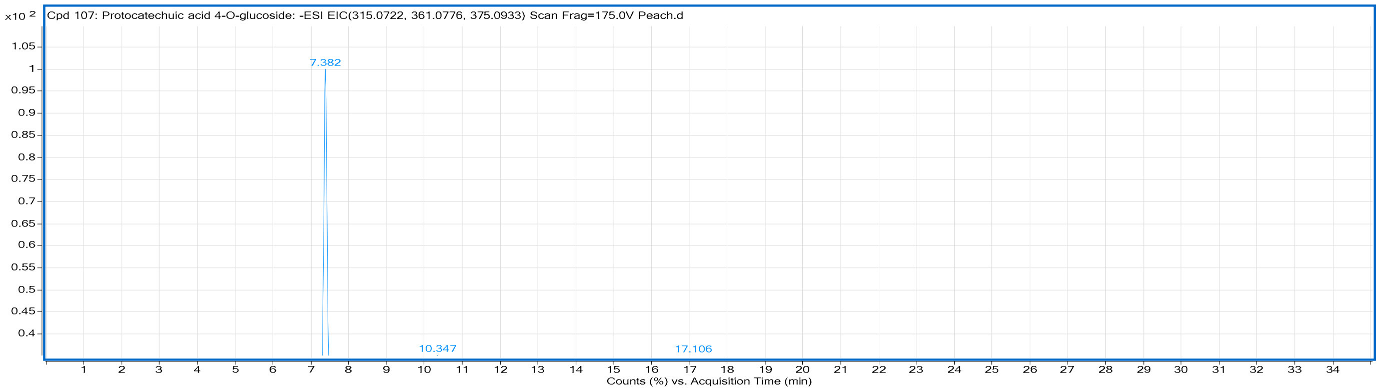
x10 2
Cpd 107: Protocatechuic acid 4-O-glucoside: -ESI EIC(315.0722, 361.0776, 375.0933) Scan Frag=175.0V Peach.d
7.382
10.347
17.106
Counts (%) vs. Acquisition Time (min)

(b)

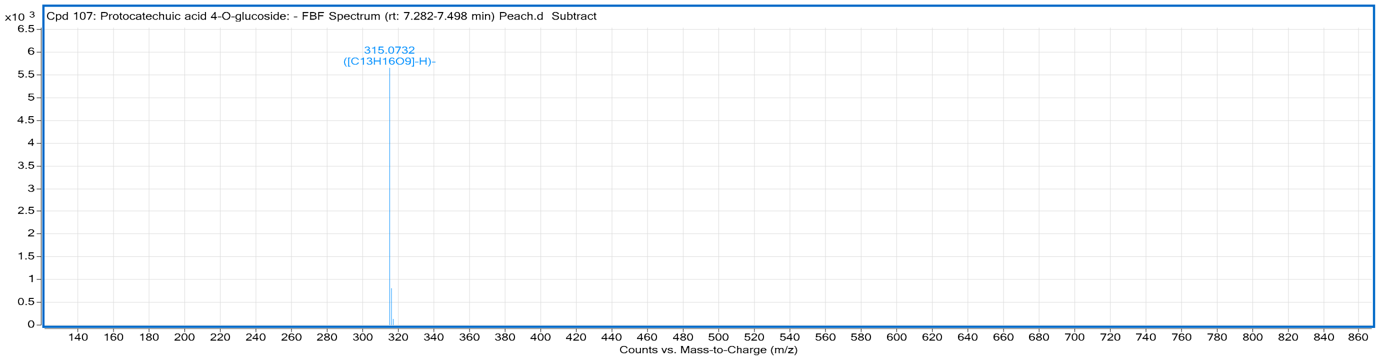
x10 3
Cpd 107: Protocatechuic acid 4-O-glucoside: - FBF Spectrum (rt: 7.282-7.498 min) Peach.d Subtract
315.0732
([C13H16O9]-H)-
Counts vs. Mass-to-Charge (m/z)

(c)

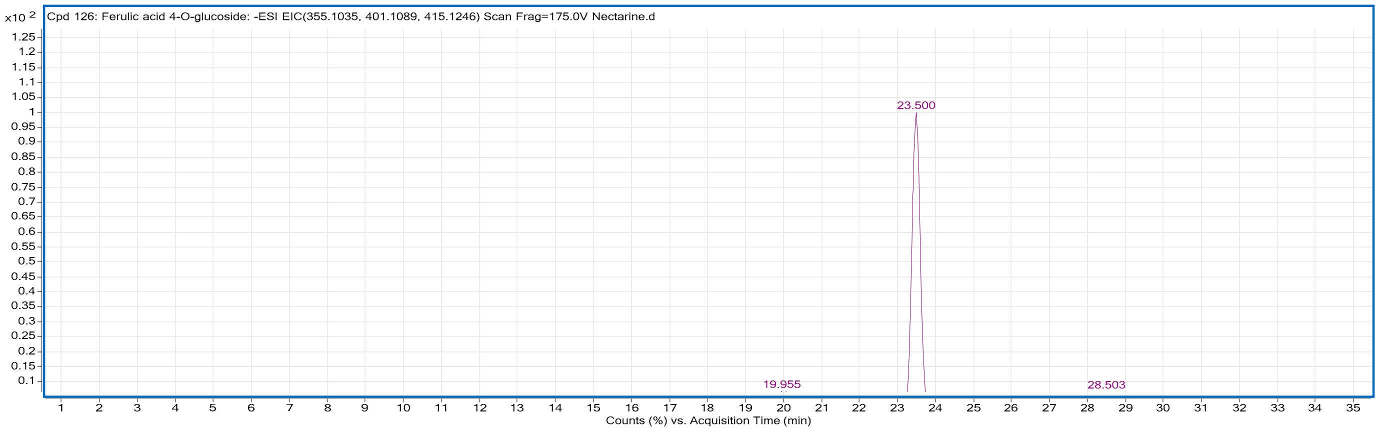
x10 2
Cpd 126: Ferulic acid 4-O-glucoside: -ESI EIC(355.1035, 401.1089, 415.1246) Scan Frag=175.0V Nectarine.d
23.500
19.955
28.503
Counts (%) vs. Acquisition Time (min)

(d)

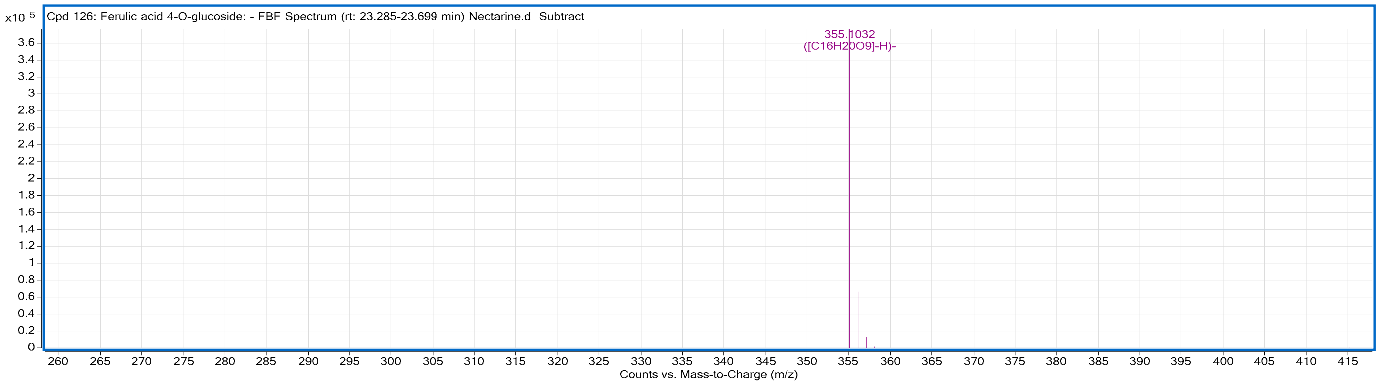
x10 5
Cpd 126: Ferulic acid 4-O-glucoside: - FBF Spectrum (rt: 23.285-23.699 min) Nectarine.d Subtract
355.1032
([C16H20O9]-H)-
Counts vs. Mass-to-Charge (m/z)

(e)

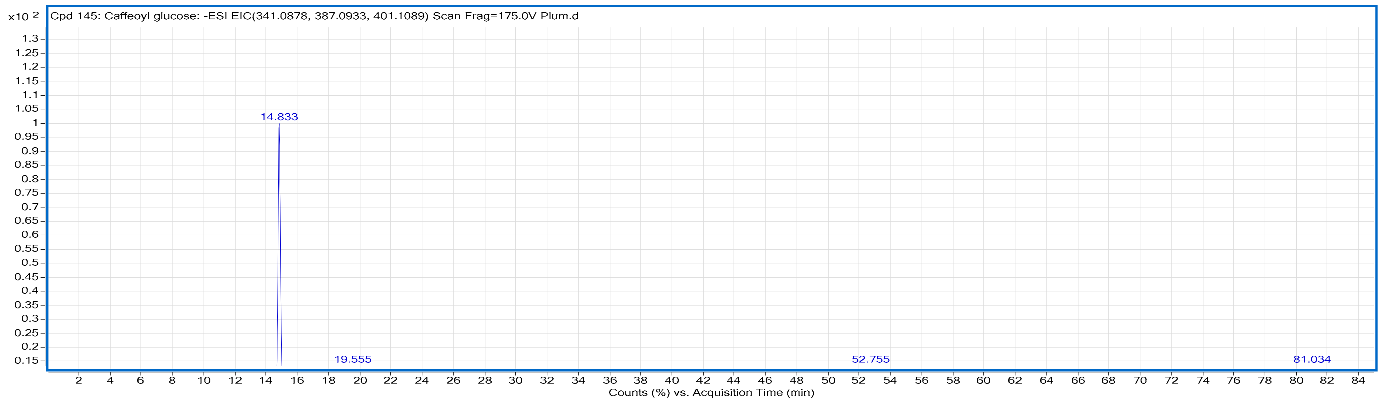
Cpd 145: Caffeoyl glucose: -ESI EIC(341.0878, 387.0933, 401.1089) Scan Frag=175.0V Plum.d
x10 2
14.833
19.555
52.755
81.034
Counts (%) vs. Acquisition Time (min)

(f)

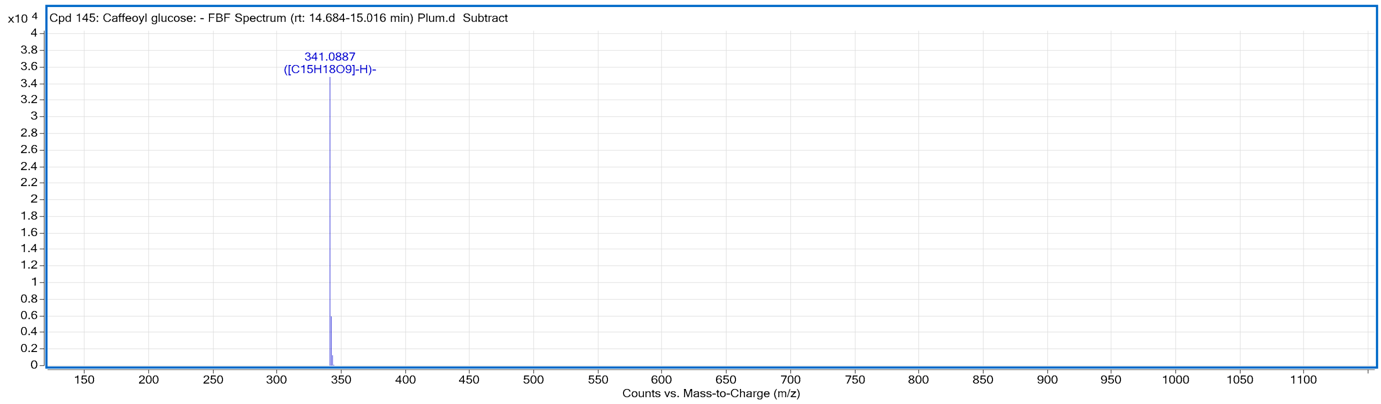
Cpd 145: Caffeoyl glucose: - FBF Spectrum (rt: 14.684-15.016 min) Plum.d Subtract
x10 4
341.0887
([C15H18O9]-H)-
Counts vs. Mass-to-Charge (m/z)

(g)

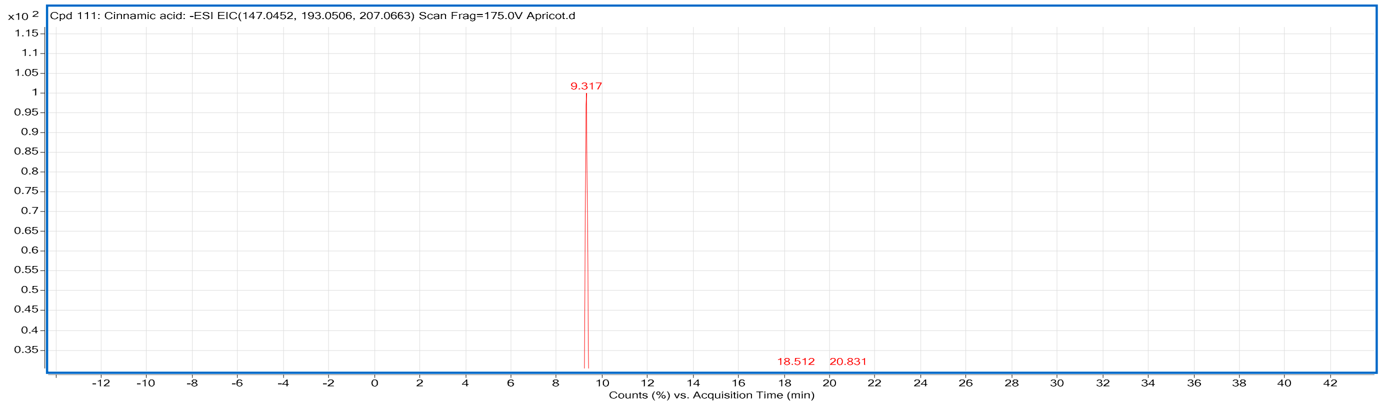
Cpd 111: Cinnamic acid: -ESI EIC(147.0452, 193.0506, 207.0663) Scan Frag=175.0V Apricot.d
x10 2
9.317
18.512
20.831
Counts (%) vs. Acquisition Time (min)

(h)

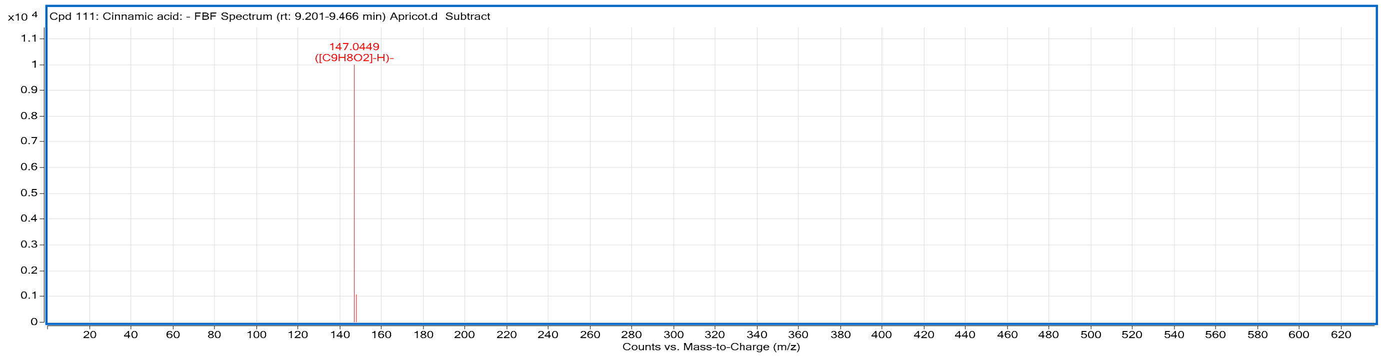
Cpd 111: Cinnamic acid: - FBF Spectrum (rt: 9.201-9.466 min) Apricot.d Subtract
x10 4
147.0449
([C9H8O2]-H)-
Counts vs. Mass-to-Charge (m/z)

Figure S2. Extracted ion chromatogram of stone fruits waste samples and their mass spectrum. (a) A chromatograph of protocatechuic acid 4-*O*-glucoside (Compound 19, Table 3), retention time (RT = 7.382 min) in the negative mode of ionization ($ESI^-/[M-H]^-$) identified and characterized in peach waste; **(b)** Mass spectra of protocatechuic acid 4-*O*-glucoside showing an observed *m*/*z* 315.0732; **(c)** A chromatograph of ferulic acid 4-*O*-glucoside (Compound 10, Table 3), retention time (RT = 23.500 min) in the negative mode of ionization ($ESI^-/[M-H]^-$) identified and characterized in nectarine waste; **(d)** Mass spectra of ferulic acid 4-*O*-glucoside showing an observed *m*/*z* 355.1032; **(e)** A chromatograph of caffeoyl glucose (Compound 4, Table 3), retention time (RT = 14.833 min) in the negative mode of ionization ($ESI^-/[M-H]^-$) identified and characterized in plum waste; **(f)** Mass spectra of caffeoyl glucose showing an observed *m*/*z* 341.0887; **(g)** A chromatograph of cinnamic acid (Compound 3, Table 3), retention time (RT = 9.317 min) in the negative mode of ionization ($ESI^-/[M-H]^-$) identified and characterized in apricot waste; **(h)** Mass spectra of cinnamic acid showing an observed *m*/*z* 147.0449.
